# Innovative approaches targeting innate immune cells to promote organ transplant tolerance

**DOI:** 10.3389/frtra.2026.1776806

**Published:** 2026-02-09

**Authors:** Chiyoshi Toyama, Angus W. Thomson

**Affiliations:** 1 Department of Surgery, Thomas E. Starzl Transplantation Institute, Pittsburgh, PA, United States; 2 Department of Immunology, University of Pittsburgh School of Medicine, Pittsburgh, PA, United States

**Keywords:** dendritic cells, immunotherapy, innate lymphoid cells, macrophages, monocytes, myeloid cells, myeloid-derived suppressor cells, natural killer cells

## Abstract

Achieving long-term allograft survival while minimizing systemic immunosuppression (IS) remains a critical unmet need in transplantation. While adaptive immunity has traditionally been the primary focus of IS therapy, innate immune cells—that include neutrophils, monocytes, macrophages, dendritic cells, myeloid-derived suppressor cells, innate lymphoid cells (ILCs), natural killer (NK) cells, and gamma delta (γδ) T cells act as key upstream orchestrators of allograft rejection and tolerance. Recent advances in single-cell RNA sequencing and spatial transcriptomics have unveiled the profound heterogeneity of these cell populations, identifying distinct regulatory subsets and novel inhibitory checkpoints. These high-resolution insights provide the scientific rationale for developing innovative precision therapies that can selectively modulate innate immune reactivity without compromising global immunity. Here, we review innovative strategies to target/amplify these mechanisms, including targeting the myeloid inhibitory checkpoints (e.g., leukocyte immunoglobulin-like receptor B; sialic acid-binding immunoglobulin-like lectin-E) to induce tolerogenic phenotypes. We further discuss the modulation of metabolic reprogramming to prevent “trained immunity” using mammalian target of rapamycin inhibitor (mTORi)-loaded nanoparticles, and the use of CRISPR (clustered regularly interspaced short palindromic repeats)/Cas9 gene editing to silence T cell costimulatory signals. We evaluate the adoptive transfer of regulatory myeloid cells, -specifically donor-derived regulatory macrophages and regulatory dendritic cells, and innate lymphoid cells in transplant recipients. Furthermore, the potential of targeting specific NK cell and ILC subsets associated with graft regulation is addressed. Collectively, these emerging approaches aim to reprogram the allograft microenvironment, offering a promising paradigm shift towards establishing transplant tolerance.

## 1 Introduction

Historically, the therapeutic control of alloreactive immune responses induced by organ transplantation advanced dramatically due to improvements in the modes of action and safety of immunosuppressive (IS) drugs, in particular development of calcineurin inhibitors (CNI) (1). This resulted in outstanding short-term (1–3 year) patient and graft survival rates (2). Such success however, has been mitigated by poor, long-term (>5-year) graft survival, patient dependence on life-long, non-specific anti-rejection medication, and the adverse, cumulative side effects of these IS agents. Further, in recent years, there has been a dearth in clinical approval of innovative and effective approaches that might bring the field closer to achieving the long-sought goal of sustained, donor-specific transplant (tx) tolerance. If successful, safe induction of tx tolerance would improve long-term graft outcomes and minimize/eliminate the need for continued, chronic IS therapy (3–5).

The effectiveness of conventional IS drugs, -principally CNI, mechanistic target of rapamycin inhibitors (mTORi), and T cell-depleting or co-stimulation blocking agents, reflects their ability to effectively suppress the adaptive immune response. Thus, antagonism of T- and B- cell dependent adaptive immunity has been studied extensively, refined in preclinical models and underpinned the successful development of organ transplantation. Increasingly, however, attention has been directed towards the roles of the innate immune system in allorecognition and immune memory (6–8), and in regulation of alloimmunity (9–12), with a focus of innate immune cells as potential targets for innovative therapeutic intervention.

While the diverse phenotypes, activation states and complex signaling pathways implemented by innate immune cells, i.e., monocytes/macrophages, dendritic cells (DCs), granulocytes, natural killer (NK) cells and innate lymphoid cells (ILCs) require further elucidation (6, 13), it is evident that these cells play important roles, not only in initiating adaptive immunity, but also in regulation of allograft rejection. Thus, importantly, the CD47- signal regulatory protein alpha (SIRPa) axis described by Dai et al. (14) represents a robust, innate immune checkpoint that modulates allorecognition by host myeloid cells. Moreover, donor-recipient mismatch at the SIRPa locus that induces innate immune activation is a determinant of human kidney tx outcome (15). Other recent studies using advanced technologies have provided insights into the contributions of innate immune cells to clinical tx outcomes (8). For instance, mRNA transcriptome analysis has revealed augmented expression of innate immune system genes during T cell-mediated human kidney tx rejection (16), while using special transcriptomics, Varin et al. (17) have identified a resident proinflammatory macrophage population (CXCL10^+^) that appears to drive renal allograft rejection. On the other hand, analyses of heart and kidney tx biopsies have shown that elevated expression of inhibitory receptors, such as sialic acid-binding immunoglobulin (Ig) -like lectins (Siglec7 and Siglec9) that are expressed on myeloid cells and NK cells and function to suppress immune responses, such as NK cell killing and T cell activity, is associated with prolonged graft survival (18).

As a further example, gene targeting and antibody (Ab)-mediated approaches have enhanced understanding of the significance of Notch pathway intercellular signaling in differentiation and fate determination of myeloid cells (macrophages and DCs) and ILCs in fine tuning the alloimmune response (19, 20). Based on studies in rodent and humanized mouse models, selective targeting of Notch-1 was shown to be a potential target for immune regulation in transplantation (21). However, evidence supporting direct targeting of Notch pathways to promote allotolerance remains limited.

Together, these findings illustrate the importance of innate immune cell populations in shaping organ tx outcomes.

Single-cell RNA sequencing (scRNA-seq) and spatial transcriptomics have revolutionized our understanding of the immune system by enabling high-resolution profiling of cellular heterogeneity and tissue-specific localization (22, 23). These technological innovations have provided unprecedented insights into innate immune cell function, under both physiological and pathological conditions. In transplantation, multi-omics approaches are increasingly being applied to dissect the complex immune landscape of graft rejection (24). Thus, for instance, recent studies have used these technologies to identify cell populations that express Fc*γ* RIII receptors (CD16; primarily NK cells, macrophages and neutrophils) that trigger functions like Ab-dependent cell-mediated cytotoxicity (25) and to elucidate how genetic variations in the myeloid checkpoint inhibitor leukocyte Ig-like receptor B3 (LILRB3) modulate myeloid cell function to impact tx outcomes (26). Building on these mechanistic insights, emerging therapeutic strategies are being developed to precisely modulate innate immunity. These include (i) Ab-mediated targeting of innate immune cells to promote their tolerogenicity (27), (ii) nano-immunotherapeutic strategies that selectively target innate immune cell populations (28, 29), (iii) gene-editing approaches employing clustered regularly interspaced palindromic repeats (CRISPR)/Cas9 technology (30), and (iv) adoptive cell therapy to enhance the numbers and function of regulatory myeloid cells (31–33), including prospective engineering of myeloid cells (34) *in vivo*.

**Figure 1:**
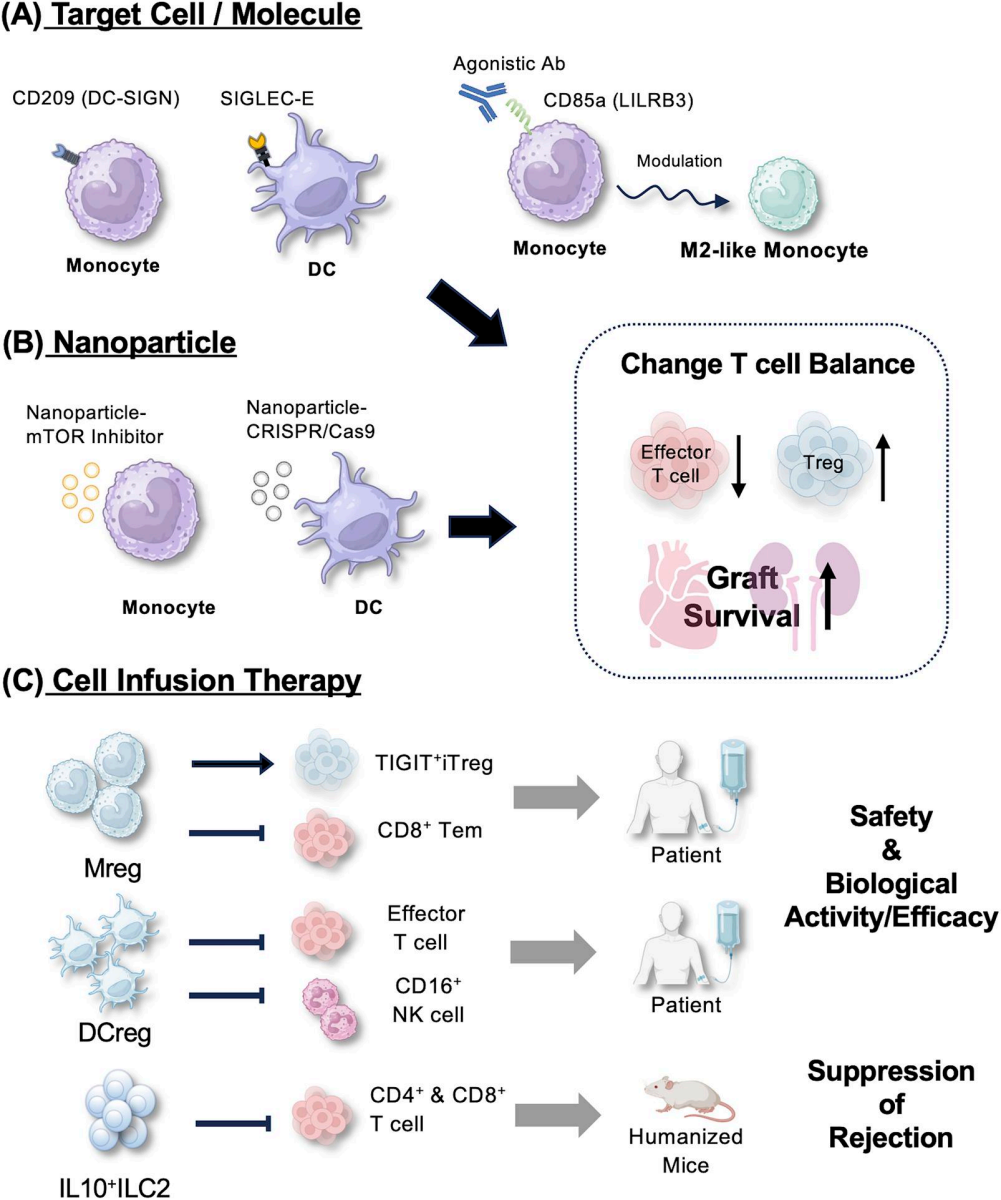
Schematic overview of innovative therapeutic strategies targeting innate immune cells to promote graft survival. **(A)** Target cell/molecule: key innate immune cells, including monocytes/macrophages, dendritic cells (DCs). Therapeutic modulation of these cell targets includes the overexpression (e.g., Siglec-E) and use of agonistic antibodies (e.g., anti-LILRB3) to reprogram monocytes/DCs toward a suppressive M2-like phenotype. **(B)** Nanoparticle-based modulation: *in vivo* therapeutic strategies include nanoparticle-based delivery of mTOR inhibitors or CRISPR/Cas9 gene-editing systems to reprogram metabolism or silence T cell costimulatory signals. **(C)** Cell infusion therapy: adoptive cell transfer of donor-derived regulatory macrophages (ddMregs), regulatory DCs (ddDCregs), or IL10 ^+^ ILC2s (allogeneic) represents an approach undergoing clinical evaluation or preclinical testing. While clinical trials have established the safety and immune modulation potential of several of these therapies, the collective goal of these interventions is to shift the immunological balance towards long-term transplant survival and tolerance.

Collectively, these approaches aim to promote tx tolerance, while minimizing the systemic side effects associated with conventional IS. In this Mini Review, we briefly discuss recent advances in targeting the innate immune system and explore therapeutic strategies tailored to exploit immunoregulatory functions of specific innate immune cell types (summarized in Figure 1; Table 1).

**Table 1:** Innovative approaches to promote the tolerogenic function of innate immune cells.

Target cell/Molecule	Therapeutic modality	Mechanism of action	Key findings/Status (species)	Reference
Monocytes/Macrophages				
DC-SIGN (CD209)	Pathway modulation (e.g., CSF1)	DC-SIGN^+^ macrophages suppress CD8^+^ T cells and promote Treg expansion	Essential for costimulation blockade-induced tolerance; IL-10 dependent (mouse)	(48)
LILRB3 (ILT5)	Agonistic mAb	Reprograms monocytes to M2-like phenotype	LILRB3 ligation induces tolerance and inhibits T cell proliferation (humanized mouse)	(27)
mTOR Pathway	mTORi-HDL nanoparticles	Inhibits aerobic glycolysis and trained immunity; promotes Ly-6C ^low^ macrophage accumulation	Prevents acute rejection; induces Treg expansion; prevents trained immunity (mouse)	(28)
C5a-C5aR1 Axis	C5aR antagonist (PMX53)	Blocks recruitment of C5aR1^+^ macrophages to the graft	Prevents macrophage accumulation and inflammation in intestinal transplant (rat)	(51)
Cellular Therapy (Mregs)	Adoptive cell transfer (doner-derived)	Suppresses effector T cells; promotes Treg induction	Minimization of immunosuppression; feasible in clinical renal transplantation (human)	(52)
Dendritic cells (DCs)				
Siglec-E	Pathway modulation (overexpression)	Inhibits NF-κB signaling; prevents DC hyperactivation and TNF-α production	Deficiency accelerates rejection; overexpression prolongs graft survival (mouse)	(18)
Costimulatory molecules (CD40/80/86)	CRISPR/Cas9 nanoparticles	Targeted gene disruption of costimulatory molecules in DCs	Prolongs allograft survival; avoids systemic toxicity (mouse)	(30)
Cell therapy (DCregs)	Adoptive cell transfer (donor-derived)	Modulates effector CD8^+^ T cell and NK cell responses	Feasible and safe; associated with lower effector T cell frequencies (human)	(72)
Natural Killer (NK) Cells				
NK Cell subsets/Ksp37	Pathway modulation (targeting cytotoxicity)	NK cells mediate Ab-independent graft injury via Ksp37 release	Identification of NK subsets associated with chronic graft dysfunction/fibrosis (human)	(74)
Myeloid-derived suppressor cells (MDSCs)				
ILT2 (LILRB1)	Pathway modulation (HLA-G interaction)	Amplifies CD11b ^+^ Gr1^+^ MDSCs; induces T cell anergy	ILT2 signaling expands MDSCs and promotes long-term allograft survival (mouse)	(86)
Donor MDSCs	Adoptive cell transfer	Donor MDSCs suppress CD8^+^ T cells and promote Treg expansion	Donor MDSCs prolong graft survival and induce recipient endogenous MDSCs (mouse)	(87)
Innate lymphoid cells (ILCs)				
ILC2 (type 2 ILCs)	Cytokine therapy (IL-33)	IL-33 expands IL-10-producing ILC2s; ILC2s promote Treg function	Prolongs islet allograft survival; ILC2 depletion abolishes protection (mouse)	(97)
Cell therapy (IL10 ^+^ ILC2)	Adoptive cell transfer (allogeneic)	Suppresses T cell-mediated cytotoxicity via IL-10 secretion and supports islet function/engraftment	Allogeneic IL-10+ ILC2s prevented islet allograft rejection and improved glucose control (humanized mouse)	(99)

Table categorizes emerging therapeutic approaches by target cell type and molecular mechanism. It highlights key findings from preclinical models (mouse, rat) and clinical studies (human), detailing the therapeutic modality and the proposed mechanism of action. CRISPR, clustered regularly interspaced palindromic repeats; CSF1, colony stimulating factor 1; DC, dendritic cell; DCreg, regulatory dendritic cell; DC-SIGN, DC-specific intercellular adhesion molecule-3-grabbing non-integrin; HDL, high-density lipoprotein; ILC, innate lymphoid cell; ILT2, immunoglobulin-like transcript 2; Ksp37, killer specific secretory protein of 37 kDa, also known as fibroblast growth factor binding protein 2; LILRB3, leukocyte immunoglobulin-like receptor B3; mAb, monoclonal antibody; MDSC, myeloid-derived suppressor cell; Mreg, regulatory macrophage; mTORi, mammalian target of rapamycin inhibitor; NF-κB, nuclear factor kappa B; NK, natural killer.

## 2 Neutrophils

Neutrophils are the first key effector innate immune cells attracted to inflammatory sites. They exhibit specialized effector functions that include neutrophil extracellular trap (NET) generation and contribute to development of a sustained inflammatory environment (35). There is evidence that they regulate acute and chronic inflammation in transplanted organs (36). Recently, NETs have been reported to regulate Kupffer cell M1 polarization during acute liver rejection (37) and DC maturation through stimulator of interferon genes (STING)-related pathways, that may promote liver rejection (38), suggesting potential for therapeutic intervention. By inhibiting NET formation through the high mobility group box 1/Toll-like receptor 4/ mitogen-activated protein kinase (HMGB1/TLR4/MAPK) signaling pathway, the natural anti-oxidant salidroside has been reported to prevent rat acute liver transplant rejection (39).

## 3 Monocytes and macrophages

In recent years, blood monocytes have been categorized into classical (CD14^++^CD16^−^), intermediate (CD14^++^CD16^+^) and non-classical (CD14 ^+^ CD16^++^) subsets based on surface markers and function (40). Within tissues, resident macrophages form a distinct compartment with unique ontogeny and roles in graft acceptance or rejection (41, 42). These cells play dual roles in organ transplantation,- as promoters of rejection and inducers of tolerance (43, 44). Regarding their role in rejection, a recent transcriptional and spatial profiling study identified a specific association between recipient-derived FcyRIIIA^+^ monocytes and the severity of intra-graft inflammation. These activated FcyRIIIA^+^ monocytes overexpressed CD47 and leukocyte Ig-like receptor (LILR) genes and increased paracrine signaling pathways, promoting T cell infiltration (25). In contrast, regulatory macrophages (Mregs) have attracted attention for their potent IS properties and potential as cellular therapeutic agents (45–47). Specific macrophage subsets have also been shown to contribute to tx tolerance. In murine models, the C-type lectin receptor DC-specific intercellular adhesion molecule-3-grabbing non-integrin (DC-SIGN)^+^ (=CD209^+^) macrophages suppressed CD8^+^ T cell proliferation and promoted the expansion of CD4^+^ forkhead box p3 (Foxp3^+^) regulatory T cells (Tregs) (48). Thus, deletion of DC-SIGN-expressing macrophages *in vivo*, interfering with their colony-stimulating factor 1 (CSF1)-dependent development, or preventing the DC-SIGN signaling pathway, abrogated co-stimulation blockade-induced heart transplant tolerance (48). Dual signaling through DC-SIGN ligands and the high mobility group box 1 protein (HMGB1)–Toll-like receptor 4 (TLR4) axis was found to be essential for the induction of IS IL-10 production (48).

Leukocyte immunoglobulin-like receptor B (LILRB) family members that are expressed mainly on myeloid cells have been viewed traditionally as inhibitory receptors. Recent studies have demonstrated the role of LILRB3 (=Ig-like transcript 5; ILT5) in regulating human monocyte/macrophage function (26). Moreover, agonistic monoclonal Abs targeting LILRB3 reprogram human monocytes toward a suppressive, anti-inflammatory/reparative M2-like phenotype, reduce T cell proliferation *in vitro* and induce immune tolerance in humanized mouse models, allowing engraftment of allogeneic cells (27). These findings suggest that LILRB3 may represent a promising therapeutic checkpoint to modulate myeloid function in transplantation. On the other hand, there is evidence (49) that engagement of LILRB3 by HLA class 1 molecules can activate the Rho-associated coiled-coil forming kinase (ROCK) signaling pathway, suggesting context-dependent activating properties. Thus, the role of LILRB signaling in the setting of alloimmunity remains incompletely understood (50) and the potential of LILRB3 targeting to promote tolerance has yet to be firmly established. Of additional note, in the context of complement signaling, macrophages expressing the complement C5a receptor 1 (C5aR1) are prevented from accumulating in intestinal grafts when the C5a-C5aR1 pathway is blocked (51). Based on these observations, effective regulation of macrophage-mediated responses in transplantation will likely require multifaceted therapeutic interventions.

Mregs can be generated *ex vivo* from circulating monocytes under specific culture conditions, such as stimulation with colony-stimulating factor 1 (CSF1; =macrophage-CSF) and IFN-*γ*, resulting in cells that produce anti-inflammatory cytokines like IL-10 and express low levels of T cell costimulatory molecules. Limited but relevant experience of allogeneic donor-derived (dd) Mreg infusion in human renal tx recipients has been documented, confirming feasibility and safety (33, 52). In these studies, the University of Regensburg group reported on >20 renal tx pts infused once with ddMreg, either 7 days before or after tx. Two patients given 7–8 × 106 Mreg/kg, 6 or 7d pre-tx were minimized to low-dose tacrolimus monotherapy within 24 weeks of tx and subsequently maintained excellent graft function.

After iv administration, most Mreg remained viable and trafficked to the liver, spleen and BM (within 30 h) (33) resembling the migration of dd regulatory DCs (ddDCreg) infused pre-tx to host lymphoid tissue of human liver tx recipients (53). No adverse events or acute rejection episodes were observed over 3-year follow-up. Mreg-based cell therapy has also been evaluated in the ONE study (54, 55), that demonstrated the safety and feasibility of administering ddMregs to kidney tx recipients. Although initial findings did not provide conclusive evidence for improved rejection control or induction of long-term tolerance, these results established a foundation for further development of Mreg (including potentially, engineered Mreg) therapies. Mregs exert their immunoregulatory effects by suppressing CD8^+^ effector memory T cell (CD8^+^ Tem) proliferation, promoting the expansion of TIGIT-expressing induced Tregs (TIGIT^+^ iTregs) (47) and modulating Ag-presenting cell function, making them promising prospective adjuncts to conventional IS agents.

Traditionally, innate immune cells were thought to lack immunological memory. However, in an important recent study, Dai et al. (7) found that murine monocytes and macrophages could acquire memory specific to MHC complex I (MHC-I) Ags and identified A-type paired Ig-like receptors (PIR-As) as the MHC-I receptors necessary for the memory response. They further showed that blocking of PIR-A binding to donor MHC-I molecules blocked memory and attenuated kidney and heart allograft rejection indicating that acquisition of alloantigen-specific memory by myeloid cells can be targeted to improve transplant outcomes. Recent studies also highlight the concept of “trained immunity,” -i.e., innate immune memory characterized by long-term epigenetic and metabolic reprogramming of monocytes and macrophages (56). This in the context of transplantation, initial pro-inflammatory stimuli, such as ischemia-reperfusion injury, can induce a “trained” phenotype in recipient myeloid cells, leading to heightened responsiveness upon secondary stimulation and potentially contributing to acute and chronic rejection (57). Crucially, the induction of trained immunity relies on a metabolic shift toward aerobic glycolysis, a process tightly regulated by the mTOR pathway (58). Addressing this mechanism, a novel therapeutic approach using high-density lipoprotein (HDL) nanoparticles (NPs) loaded with mTOR inhibitors (mTORi-HDL) has been developed (28). These NPs are preferentially taken up by myeloid cells, especially macrophages in mouse heart allografts and systemically. mTORi-HDL treatment of the graft recipients enhances the accumulation of Ly-6C^low^ macrophages, that promote allograft tolerance (59). Inflammatory cytokines such as tumor necrosis factor (TNF)-*α* and IL-6, as well as lactate production, are significantly reduced, suggesting macrophage metabolic reprogramming toward an anti-inflammatory phenotype.

## 4 DCs

BM-derived DCs are the most proficient Ag-acquiring, -processing and -presenting cells and play a central role in initiation and regulation adaptive immune responses. They display inherent tolerogenic properties (60). Mechanisms whereby tolerogenic DCs mediate their immunoregulatory functions, including suppression of T effector cells, differentiation of CD4 Tregs and induction of regulatory properties in B cells, NK cells and CD8T cells have been reviewed (61–63). Moreover, Marin et al. (64) have reported that the tolerogenic function of DCs is linked intrinsically to distinct metabolic programs, making cell metabolism a central driver of DCreg function.

DCregs can be generated *in vitro* from circulating monocytes using pharmacologic or biologic agents that promote their tolerogenicity (65). When adoptively transferred to allograft recipients in preclinical models, these cells can promote tx tolerance (31, 66). Thus, DCs have emerged both as therapeutic targets and tools for promoting tx tolerance (67). NP-mediated delivery of CRISPR/Cas9 components enables *in situ* gene editing in DCs (30). Thus, in a murine skin tx model, iv injection of NPs carrying Cas9 mRNA and guide RNA targeting CD40 (CLANmCas9/gCD40) successfully induced Cas9 expression and CD40 gene knockout in DCs. While this approach shows promise for controlling rejection, further safety assessment is essential.

Siglec-E is an inhibitory receptor, found primarily on myeloid cells that acts as a negative regulator of inflammation by binding to sialic acid on other cells (68, 69). It regulates DC activation and T cell-mediated rejection in a mouse heart tx model (18). Thus Siglec-E deficiency enhances DC activation and inflammatory cytokine (IL-6, TNF-α, IL-18) production, accelerating acute rejection (18). In the same study, human homologs of Siglec-E, namely Siglec7 and Siglec9, were downregulated in patients with rejection and associated with decreased graft survival, indicating that targeting of these receptors may help suppress DC activation and promote tolerance.

DCregs can be generated *in vitro* from BM cells or peripheral blood monocytes using immunomodulatory agents, such as vitamin D3 or IL-10 (70). Compared to mature DCs, DCregs exhibit low expression of MHC class II and costimulatory molecules (CD80, CD86, CD40), while expressing higher levels of the inhibitory ligand programmed death-ligand 1 (PD-L1). DCregs are also resistant to maturation upon TLR ligation or inflammatory cytokine stimulation.

In a non-human primate (NHP) kidney transplant model, pre-tx infusion of ddDCregs significantly prolonged graft survival in animals receiving minimal IS therapy (71). In clinical studies of live donor liver transplantation, pre-tx administration of ddDCregs has proven feasible and safe and is associated with a reduction in effector T cells (T-bet ^+^ Eomesodermin ^+^ CD8^+^ T cells) and CD16^high^ NK cells (72). An increase in circulating, tolerogenic CD141 ^+^ CD163^+^ DCs, which may contribute to immunoregulation, was also observed. The long-term impact of these findings on graft survival is currently under investigation.

## 5 NK cells

In transplantation, NK cells contribute to both rejection and tolerance, depending on the context (73). Their activation is regulated by a balance of inhibitory and activating receptors, such as transmembrane killer-cell Ig-like receptors (KIRs) and NK group 2 member D (NKG2D), interacting with MHC class I molecules.

Recent studies of human allograft tissues have documented NK cell infiltration in acute rejection, with a particular increase in CD56^low^ NK cells associated with cytotoxic potential (74). In murine models, NKG2D expression increases over the course of ischemic injury, the extent of which is reduced by adoptive transfer of NKG2D^−/−^ NK cells, or through blockade of NKG2D (75). Furthermore, alloreactive adaptive NK cell subsets have been linked to microvascular inflammation in renal transplantation (76). Reinforcing this observation, recent spatial transcriptomics analysis has identified FcyRIII^+^ NK cells as key drivers of microvascular inflammation, acting alongside monocytes to recruit T cells via paracrine signaling (25). The development of novel anti-CD38 mAbs represents an important innovation in modulation of NK cell activity. As reported recently (77), encouraging effects have been achieved targeting CD38 in a phase II trial to inhibit kidney graft injury caused by alloantibodies and NK cells.

Under certain conditions however, NK cells may promote tolerance by eliminating donor Ag-presenting cells, thereby reducing T cell priming (78). Therapeutically, blockade of activating NK receptors and augmentation of inhibitory signaling are being investigated as strategies to limit NK cell activity (74). Potentially, expansion of tolerogenic NK subsets could be harnessed to promote graft acceptance. Overall, the dual roles of NK cells in mediation of rejection and regulation make complex but potentially promising targets for transplant immunotherapy (79).

## 6 MDSCs

MDSCs are a heterogeneous population of IS cells that develop from immature myeloid cells under inflammatory conditions (80). They are broadly classified into two main subsets: polymorphonuclear (PMN)-MDSCs and monocytic (M)-MDSCs that exhibit distinct IS mechanisms. Thus, PMN-MDSCs primarily produce reactive oxygen species and M-MDSCs mediate suppression via nitric oxide and arginase-1 pathways (81, 82). Understanding the biology/functional heterogeneity of MDSCs is critical for their potential therapeutic exploitation (83, 84).

In transplantation, MDSCs have attracted attention due to their synergistic interactions with Tregs in promoting and maintaining rodent kidney allograft tolerance (85). Engagement of the inhibitory receptor LILRB1 (ILT2 or CD85j) that suppresses T cell activation, by its principal ligand HLA-G, expands MDSCs with increased suppressive activity (86). Moreover, adoptive transfer of these MDSCs generated via the ILT2–HLA-G axis promotes long-term skin allograft survival in mice. These observations suggest that induction of MDSCs using ILT2-HLA-G might be a valuable approach to suppression of rejection. Notably, donor-derived MDSCs prolong mouse cardiac allograft survival in a donor-specific manner by inducing recipient endogenous MDSCs and suppressing effector T cell responses (87). Human MDSCs represent a promising immunoregulatory cell population that inhibits xenogeneic graft-vs.-host disease in humanized NOD/SCID/IL2-R*γ*_c_^−/−^ mouse models (88, 89)**.**

Recent research has helped to elucidate the complexity of MDSC biology in transplantation (84). Differentiating between PMN- and MDSC subsets has revealed their distinct roles in immune suppression (90). Notably, interactions between MDSCs and other innate immune cells, including DCs and NK cells, are emerging as important modulators of graft outcomes. Recent reports have also focused on a distinct metabolic phenotype underlying the differentiation of MDSCs in an inflammatory microenvironment, representing a regulatory target (91). Moreover, as with other regulatory immune cells, exosome products of MDSCs may play key roles in mediating their IS functions (92). These insights suggest that multifaceted approaches targeting MDSC subsets and their crosstalk with other immune cells may be necessary to harness their full therapeutic potential in transplantation.

## 7 ILCs

Based on their expression of transcription factors and cytokine profiles, ILCs are classified into three main groups: ILC1, ILC2, and ILC3, that functionally mirror Th1, Th2, and Th17 subsets, respectively (93, 94). Among these, type 2 ILCs (ILC2s), are the best-defined, depend on the transcription factor GATA binding protein 3, produce archetypal type 2 cytokines (such as IL-4, IL-5, IL-9 and IL-13) and are involved in allergic inflammation and anti-parasitic responses (95, 96). In a murine islet allograft model, IL-33 treatment significantly prolongs tx survival, increasing the frequency of ILC2s and Tregs in the spleen, kidney, and transplanted islets (97). ILCs are a key source of IL-10 (98) production that is critical for the IS function of ILC2s. Co-culture with IL-33 and IL-2 complexes significantly increases the proportion of IL-10–producing ILC2s and their adoptive transfer prolongs islet allograft survival, in an IL-10-dependent manner. Recent reports have shown that human IL-10 ^+^ ILC2s have therapeutic potential in islet allograft transplantation (99), xenogeneic graft-vs. host disease (100) and other immune-mediated disorders (101).

Although ILC2s have gained most attention for their regulatory potential, other ILC subsets may also influence tx outcome. Thus, recent studies by Kojima et al. (102) have revealed a novel, IFNg-mediated cytoprotective role of both recipient and donor ILC1s against ischemia-reperfusion injury in murine liver transplantation. In addition, ILC3s have been associated with successful human intestinal transplantation. Thus, serial monitoring revealed that in healthy allografts, protective ILC3s repopulated by 2–4 weeks post-tx, whereas in rejecting grafts they remained diminished (103).

Detailed mechanistic insights, potential augmentation and functional roles of these protective ILCs in preclinical tx models remain to be elucidated. Future studies should explore the therapeutic manipulation of ILCs, such as via cytokine therapy or *ex vivo* expansion strategies, that may help promote tolerance in clinical settings.

## 8 γδ T cells

Gamma delta (γδ) T cells are a unique population of lymphocytes that bridge innate and adaptive immunity. Various subsets may be considered components of the innate immune system (104). In particular, IL-17A + γδ T cells play a role in early stages of inflammation (105). In a murine skin transplant model, they contribute to the accumulation of mature DCs in draining lymph nodes, thereby regulating αβ T cell function and facilitating cross-priming of CD8^+^ T cells (106, 107). In a recent report, IL-17A γδ T cells, together with monocytes, have been associated with rapid alloimmune reactivity following mouse vascularized composite allograft (VCA) transplantation (108). This suggests that specific targeting of IL-17A γδ T cells and classical monocytes, that are not targeted specifically by current immunosuppressants, may control VCA rejection. Regulatory γδ T cells are a rare population of immunosuppressive γδ T cells. They use direct cell-to-cell interaction or secrete inhibitory cytokines such as IL-10 and TGF-β to mediate their functions (109, 110). To assess their full potential in the context of transplantation, a deeper comprehension of γδ T cell development and plasticity is essential (111).

## 9 Conclusions

Innate immune cells, including neutrophils, monocytes/macrophages, DCs, MDSCs, NK cells, ILCs, and γδ T cells play diverse and critical roles in both graft rejection and tolerance. While these cells sense graft injury and initiate inflammation, their functions can potentially be harnessed or modulated to promote IS and tolerance. Recent technological advances—such as sc RNA sequencing, spatial transcriptomics, nanomedicine, and CRISPR/Cas9—have enabled unprecedented resolution in characterizing the heterogeneity and functional states of innate immune cells. These tools have provided the foundation for precise analysis of cellular populations, assessment of cell enrichment and activity, and the development of cell-targeted therapeutic strategies. Since each innate immune cell population comprises distinct subsets, a key challenge to clinical translation is identification of the most effective and stable cell type that can most easily be safely programmed/augmented *in vivo* in conjunction with conventional or emerging immunosuppressive agents that primarily target adaptive immune cells. Deeper understanding of mechanisms of action of the most promising strategies and confirmation of their relevance to clinical outcomes in transplantation are needed. Nevertheless, immunotherapies that selectively target/program specific innate immune cell subsets hold promise for reducing/minimizing the adverse effects of global IS, while achieving long-term allograft acceptance. Therapeutic strategies designed to reprogram specific immune cell functions may pave the way for personalized tx medicine and significantly improve patient outcomes.

## Glossary

Ab: antibody
Ag: antigen
CLANmCas9/gCD40: Cas9 mRNA and guide RNA targeting CD40
CD8+ Tem(s): CD8 + effector memory T cell(s)
C5aR1: C5a receptor 1
CSF1: colony-stimulating factor 1
CNI: calcineurin inhibitor
CRISPR: clustered regularly interspaced short palindromic repeats
DC(s): dendritic cell(s)
DCreg(s): regulatory DC(s)
DC-SIGN: DC-specific intercellular adhesion molecule-3-grabbing non-integrin
dd: donor-derived
Foxp3: forkhead box p3
γδ T cell(s): Gamma delta T cells
GATA3: GATA-binding protein 3
HDL: high-density lipoprotein
HMGB1: high mobility group box 1 protein
HMGB1/TLR4/MARK: high mobility group box 1/Toll-like receptor 4/ mitogen-activated protein kinase
ILC(s): innate lymphoid cell(s)
IS: immunosuppressive or immunosuppression
ILC1(s): type 1 ILC(s)
ILC2(s): type 2 ILC(s)
ILC3(s): type 3 ILC(s)
ILT: immunoglobulin-like transcript
KIR(s): killer-cell immunoglobulin-like receptor(s)
LILR(s): leukocyte immunoglobulin-like receptor(s)
MDSC(s): myeloid-derived suppressor cell(s)
M-MDSC(s): mononuclear MDSC(s)
Mreg(s): regulatory macrophage(s)
mTORi: mechanistic target of rapamycin inhibitor
NET(s): neutrophil extracellular trap(s)
NHP: non-human primate
NK cell(s): natural killer cell(s)
NKG2D: NK group 2 member D
NP(s): nanoparticle(s)
PD-L1: programed death-ligand 1
PMN-MDSC(s): polymorphonuclear-MDSC(s)
ROCK: Rho-associated coiled-coil forming kinase
SIGLEC: sialic acid-binding immunoglobulin-like lectin
scRNA-seq: single-cell RNA sequencing
SIRPa: Signal regulatory protein alpha
TCMR: T cell–mediated rejection
TIGIT^+^ iTreg(s): T cell immunoreceptor with Ig and immunoreceptor tyrosine-based inhibitory motif domains-expressing induced Treg(s)
TLR(s): Toll-like receptor(s)
tx: transplant
